# A Hybrid Compact Convolutional Transformer with Bilateral Filtering for Coffee Berry Disease Classification

**DOI:** 10.3390/s25133926

**Published:** 2025-06-24

**Authors:** Biniyam Mulugeta Abuhayi, Andras Hajdu

**Affiliations:** 1 Department of Information Technology, University of Gondar, Gondar P.O. Box 196, Ethiopia; 2 Department of Data Science and Visualization, Faculty of Informatics, University of Debrecen, Egyetem ter 1, 4032 Debrecen, Hungary; hajdu.andras@inf.unideb.hu

**Keywords:** compact convolution transformers, bilateral filtering, coffee berry disease

## Abstract

Coffee berry disease (CBD), caused by Colletotrichum kahawae, significantly threatens global Coffee arabica production, leading to major yield losses. Traditional detection methods are often subjective and inefficient, particularly in resource-limited settings. While deep learning has advanced plant disease detection, most existing research targets leaf diseases, with limited focus on berry-specific infections like CBD. This study proposes a lightweight and accurate solution using a Compact Convolutional Transformer (CCT) for classifying healthy and CBD-affected coffee berries. The CCT model combines parallel convolutional branches for hierarchical feature extraction with a transformer encoder to capture long-range dependencies, enabling high performance on limited data. A dataset of 1737 coffee berry images was enhanced using bilateral filtering and color segmentation. The CCT model, integrated with a Multilayer Perceptron (MLP) classifier and optimized through early stopping and regularization, achieved a validation accuracy of 97.70% and a sensitivity of 100% for CBD detection. Additionally, CCT-extracted features performed well with traditional classifiers, including Support Vector Machine (SVM) (82.47% accuracy; AUC 0.91) and Decision Tree (82.76% accuracy; AUC 0.86). Compared to pretrained models, the proposed system delivered superior accuracy (97.5%) with only 0.408 million parameters and faster training (2.3 s/epoch), highlighting its potential for real-time, low-resource deployment in sustainable coffee production systems.

## 1. Introduction

Coffee stands as the second largest commodity traded in international markets and one of the most valuable agricultural commodities in history, with far-reaching impacts from farmers to consumers worldwide [1]. The coffee industry serves as a vital source of livelihood for numerous communities across the globe and plays a significant role in the national economies of various countries. Ensuring the sustainability of the industry and providing coffee farmers with consistent profits requires the implementation of cultivation practices that are more environmentally conscious [2].

Coffee berry disease (CBD), caused by the fungus Colletotrichum kahawae, is one of the most severe threats to coffee production, particularly in East Africa. The disease manifests as anthracnose lesions on coffee berries, leading to premature fruit drop and substantial yield losses, sometimes reaching 80% in severely affected regions [3,4]. The spread of CBD is influenced by environmental factors such as high humidity and rainfall, which create optimal conditions for fungal proliferation [5]. Additionally, genetic susceptibility plays a role, as Arabica coffee (*Coffea arabica*) is highly vulnerable due to its limited resistance to *C. kahawae* [6]. Management strategies for CBD include breeding resistant varieties, applying fungicides, and implementing cultural practices like pruning and sanitation to reduce pathogen load [7]. Recent advancements in machine learning and image processing have also enabled early detection, improving disease monitoring and control [8,9].

Coffee berry disease (CBD) poses a critical challenge to *Coffea arabica* plants, significantly impacting the coffee industry worldwide [10]. Traditional methods for detecting coffee diseases often rely on manual observation by farmers or experts. These approaches are inherently subjective, time-consuming, and susceptible to human error. Furthermore, they may not facilitate early detection, which is crucial for effective disease management and preventing widespread outbreaks [11,12]. The heavy reliance on chemical treatments for disease control also raises significant concerns regarding environmental sustainability and the long-term viability of agricultural practices. Moreover, the practical implementation of automated disease detection systems in agricultural settings often requires models that are not only accurate but also computationally efficient, capable of operating with limited data and resources.

Modern approaches are increasingly leveraging advancements in artificial intelligence (AI), particularly deep learning and machine learning, to automate disease detection and classification. These methods offer the potential for more objective, efficient, and scalable solutions compared to traditional techniques. Deep learning models, such as Convolutional Neural Networks (CNNs), have demonstrated remarkable success in processing and analyzing images for various disease detection tasks [13,14].

Deep learning models have been extensively applied to coffee disease detection, with Convolutional Neural Networks (CNNs) being particularly effective for automatic feature extraction from images of coffee leaves. Numerous studies have demonstrated the efficacy of CNNs and transfer learning approaches in accurately classifying various coffee leaf diseases [15,16]. EfficientNet models, including EfficientNetB0 and EfficientNetB1, have gained prominence for their balance between accuracy and computational efficiency, often outperforming other architectures like VGG16 and ResNet50. Lightweight models such as MobileNetV2, frequently combined with Support Vector Machines (SVMs), have also shown promising results in resource-constrained environments [17]. Transfer learning has further enhanced model performance by enabling deep networks to generalize effectively with limited training data [18]. Ensemble techniques, such as combining multiple EfficientNet variants, have improved robustness, while hybrid models integrating CNNs with SVMs or visual transformers have further refined classification performance [19]. Additionally, explainability methods like Class Activation Maps (CAMs) and Grad-CAM have played a vital role in interpreting model decisions, offering insights into how deep learning models identify disease patterns in coffee leaves [20].

While the aforementioned methods have proven valuable in addressing various plant disease detection challenges, a notable gap exists in the specific application of advanced deep learning techniques to coffee berry diseases. Existing research has predominantly focused on diseases affecting coffee leaves, often overlooking the direct impact of CBD on the berries themselves. Given that coffee berry disease directly affects both the quality and quantity of coffee yields, its accurate and early detection is crucial. Furthermore, many existing deep learning models are computationally intensive and require large datasets, posing challenges for deployment in agricultural settings with limited resources and data availability. These urgent challenges underscore the necessity for automated systems specifically designed to effectively identify and classify coffee berry diseases, thereby improving harvest yields and minimizing losses caused by this devastating pathogen. The development of robust deep learning models for this task, however, requires large, high-quality datasets of coffee berries, which can be challenging to acquire. Therefore, developing models that can achieve high accuracy with smaller datasets and fewer parameters is crucial for practical implementation.

To address this gap and the practical constraints of agricultural deployment, this study proposes a novel pipeline for the automated classification of healthy and CBD-affected coffee berries. Our approach leverages a Compact Convolutional Transformer (CCT) model for robust feature extraction, enhanced by preprocessing techniques including bilateral filtering and color segmentation to improve image quality and feature discriminability. The extracted features are then classified using a Multilayer Perceptron (MLP) optimized with early stopping and regularization techniques. Furthermore, the generalizability of the CCT-extracted features is evaluated using various optimized machine learning classifiers, including Support Vector Machines (SVMs), K-Nearest Neighbors (KNN), Decision Trees (DTs), and Random Forests (RFs). The performance of the proposed model is also compared against well-established pretrained architectures to highlight its effectiveness and efficiency in terms of parameter size and training time. The contributions of this work include the following:

- **Development and Evaluation of a Compact CCT Model:** A novel Compact Convolutional Transformer (CCT) model was developed and evaluated for automated coffee berry disease classification, achieving state-of-the-art accuracy with a reduced number of parameters.
- **Dedicated Coffee Berry Dataset and Targeted Research:** This study utilized a specific dataset of coffee berry images, directly addressing the research gap in the detection of this critical disease affecting the fruit.
- **Enhanced Performance through Preprocessing:** The implementation of bilateral filtering and color segmentation significantly improved image quality and subsequently enhanced the performance of various classifiers.
- **Superior Efficiency and Robust Feature Representation:** Comprehensive comparative analysis demonstrated the proposed model’s higher accuracy, smaller size, and faster training compared to pretrained models. Furthermore, the CCT-extracted features proved robust and versatile across multiple optimized machine learning classifiers, highlighting the model’s potential for resource-limited deployment.

## 2. Literature Review

The study by [21] proposed a deep learning approach for real-time coffee leaf disease identification, employing transfer learning models such as ResNet101, Xception, CoffNet, and VGG16. These models demonstrated high accuracy and processing speed, making them suitable for agricultural applications. Similarly, reference [8] investigated the EfficientNetB0 model for classifying coffee leaf diseases using a dataset from Kaggle, achieving a high accuracy of 96% and emphasizing early diagnosis for sustainable farming.

A Deep-Siamese CNN-based approach for diagnosing Mycosphaerella Coffeeicola disease in coffee foliage was introduced in [22], achieving an accuracy of 96.89%, highlighting its potential for precision agriculture. Furthermore, reference [23] proposed SUNet, a hybrid deep learning model combining U-Net and SegNet for effective disease detection. The model leveraged VGG16 for feature extraction and Mask R-CNN for precise localization of disease spots. In another study, reference [24] developed the CoffeeNet model utilizing an improved CenterNet approach with a spatial-channel attention strategy based on ResNet-50, which achieved 98.54% classification accuracy under realistic agricultural conditions.

Mobile applications have also been explored for coffee disease classification. A study by [25] introduced a mobile app employing DenseNet, achieving 99.57% training accuracy. The integration of GPS functionality in the app enhances disease classification by providing location-specific data for efficient management. Similarly, reference [26] utilized Convolutional Neural Networks (CNNs) to classify coffee leaf rust (CLR) using a dataset of 1365 images, obtaining 98.89% accuracy, making it a viable real-time disease management tool.

Other research efforts have explored transfer learning and data augmentation techniques to enhance classification accuracy. For instance, reference [27] employed deep learning models like ResNet50 and MobileNet for coffee leaf disease classification, achieving high accuracy rates. A review conducted in [28] highlighted the effectiveness of frameworks such as MobileNet and ResNet50 in achieving detection accuracies ranging from 82% to 99.3%, reinforcing their potential in agricultural settings.

Alternative methods for disease classification have also been examined. The study by [29] introduced an enhanced multivariance product representation (EMPR) approach utilizing CNN architectures like VGG16, which achieved 96% accuracy. Additionally, deep learning models, including CNNs and transfer learning models like MobileNet and ResNet50, have been widely used, achieving classification accuracies between 98% and 100% [30]. These models are often trained on datasets collected from Mendeley, utilizing visualization techniques such as Grad-CAM and Grad-CAM++ to generate heat maps that highlight key classification regions [31,32].

Machine learning techniques have also been applied to coffee disease classification. Support Vector Machines (SVMs) and Random Forest algorithms have been utilized, obtaining accuracy between 81.03% and 100% [33]. These methods have been tested on datasets such as JMuBEN, providing an alternative to deep learning approaches for automated disease identification.

In addition to computer vision techniques, other computational approaches have been explored. A study by [34] proposed a method combining wireless sensor networks, remote sensing, and deep learning to detect coffee leaf rust, achieving an F1-score of 0.775. Meanwhile, reference [35] introduced an expert system for diagnosing coffee diseases in their early stages using computational methods. Texture-based statistical approaches, including Local Binary Patterns (LBPs), were investigated in [36], achieving a kappa coefficient of 0.97 and sensitivity of 0.98.

Semantic segmentation techniques have also been employed for coffee disease identification. An Android application designed for identifying four types of coffee leaf diseases and severity estimation using ResNet50 achieved 97% accuracy [37]. A case study by [38] explored few-shot learning for classification and severity estimation, reporting a 96% accuracy rate. Additionally, reference [39] proposed a deep learning model to identify coffee wilt disease using a dataset of 4000 images, achieving a test accuracy of 97.9%.

Moreover, a CNN-based approach integrating feature concatenation of ResNet and GoogleNet was introduced by [40] to classify and detect seven types of coffee diseases, including coffee berry disease. This method achieved an impressive accuracy score of 99.08%, demonstrating the effectiveness of ensemble techniques in improving classification performance. Similarly, Convolutional Neural Networks (CNNs) remain foundational to many image classification tasks due to their ability to perform hierarchical feature extraction through convolutional, pooling, and fully connected layers [41,42]. Modern advances such as ResNet and DenseNet have addressed challenges like vanishing gradients and overfitting, further improving generalization performance in complex datasets, including medical and agricultural images.

Recently, Compact Convolutional Transformers (CCTs) have emerged as a hybrid architecture that integrates convolutional operations with transformer layers to enhance performance, especially in data-scarce environments. These models use convolutional tokenization to capture local spatial features and leverage transformer blocks to model long-range dependencies [43,44]. Enhanced CCT variants, such as Improved CCT (ICCT), refine positional encoding and feed-forward mechanisms, resulting in higher classification accuracy with reduced computational overhead [45]. In agricultural contexts, CCTs have demonstrated success in anomaly detection and disease classification through encoder–decoder designs, while optimization techniques like Sharpness-Aware Minimization (SAM) and Gradient Centralization (GC) have been applied to further boost training stability and performance.

## 3. Materials and Methods

### 3.1. Dataset Details and Preprocessing

**Figure 1:**
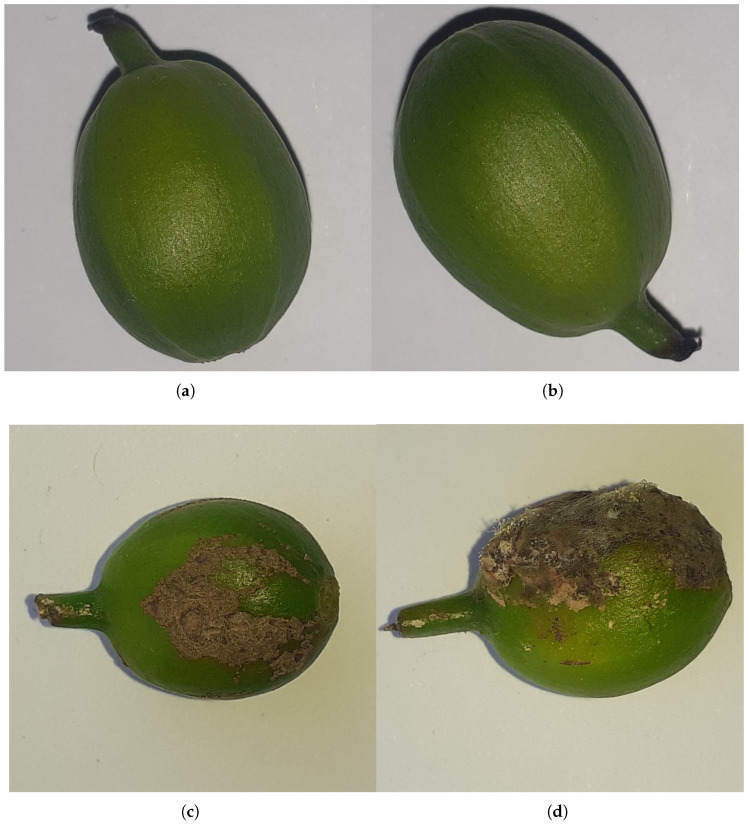
Sample images of coffee berries illustrating both healthy and diseased conditions. (**a**,**b**) show healthy coffee berries with uniform color and intact surface. (**c**,**d**) display diseased coffee berries exhibiting visible symptoms such as discoloration, deformation, or fungal infection.

The dataset used in this study was obtained from the University of Gondar’s Plant and Veterinary Science College. It consists of 1737 images of coffee berries, classified into two categories: healthy coffee berries and those affected by coffee berry disease, as illustrated in Figure 1. The images were captured after the berries were collected from the field using a 15-megapixel camera positioned directly above the samples under natural lighting conditions. The captured berries represent a variety of disease stages, from early signs of infection to severely affected fruits, and include coffee berries at different maturity levels, as the collection was performed randomly across the field. Data partitioning: The dataset was divided into training, validation, and testing sets with a ratio of 60:20:20, respectively. This partitioning was performed using the train_test_split function from Scikit-Learn to ensure a comprehensive and unbiased evaluation of the model’s performance on unseen data. The balance of categories within each split was considered to provide a reliable assessment of the model’s generalization capability across both healthy and diseased berries. Image preprocessing: All images underwent a resizing process to a uniform dimension of 128 × 128 pixels using the target_size parameter within Keras ImageDataGenerator. The RGB color mode was maintained to align with standard practices in deep learning for image analysis. Label encoding: Categorical labels (healthy and diseased) were transformed into a numerical format suitable for model training through one-hot encoding. This conversion enables the use of categorical cross-entropy loss, which is appropriate for multi-class classification tasks. Image denoising: Bilateral filtering was applied as a denoising technique specifically for the Compact Convolutional Transformer (CCT) model. This choice was motivated by bilateral filtering’s effectiveness in reducing noise while preserving critical edge details in RGB images. Unlike conventional smoothing filters like Gaussian or median filtering, bilateral filtering avoids excessive blurring of important structural information. This is particularly advantageous for the CCT architecture, where convolutional layers extract local spatial features and transformers capture long-range dependencies. By employing bilateral filtering, we aim to ensure that the input features presented to the model are cleaner and retain essential details, thereby enhancing the feature extraction process in the convolutional layers, leading to improved classification accuracy and increased robustness to variations in lighting and minor image perturbations. Data diversity and augmentation: The dataset, while originating from a single geographical location, exhibits some degree of natural diversity in terms of disease stage and maturity of the coffee berries due to the random collection process and varying degrees of natural light exposure during image capture. However, techniques such as Generative Adversarial Networks (GANs) for data augmentation were not employed in this study. Our primary objective was to evaluate the performance of the proposed model on a limited real-world dataset without introducing synthetically generated images. Future work may explore the impact of data augmentation techniques on the model’s generalization capabilities.

### 3.2. Proposed Model Architecture

This section provides a detailed overview of the architectural components and their integration, forming a robust framework for feature extraction, fusion, and classification using the CCT model. Our methodology proposes a novel Compact Convolutional Transformer (CCT) model for image classification by integrating convolutional feature extraction with transformer-based feature processing. The model consists of two parallel convolutional branches that extract hierarchical features, which are then concatenated and processed through a transformer encoder before classification. This hybrid architecture aims to leverage the strengths of both convolutional networks and transformers to improve classification accuracy and robustness. The integration of convolutional feature extractors with a transformer-based encoder offers several key advantages: feature enrichment: By combining feature representations from both convolutional branches, the model captures diverse spatial patterns and enhances contextual understanding. The convolutional layers specialize in local feature extraction, while the transformer encoder models long-range dependencies, leading to a more comprehensive feature representation. Improved performance: The fusion of convolutional and transformer-based features significantly enhances classification accuracy. The transformer’s attention mechanism refines the extracted features, improving the model’s ability to differentiate between classes. Increased model capacity: The concatenation of feature maps expands the model’s capacity to learn complex patterns, making it particularly effective for large and diverse datasets. This increased capacity improves generalization and adaptability to various image classification tasks. Robustness to variations: The convolutional layers provide invariance to small spatial transformations, while the transformer’s self-attention mechanism enables better adaptation to variations in scale, orientation, and lighting conditions. This robustness is essential for handling diverse real-world image datasets.

### 3.3. Model Training

**Figure 2:**
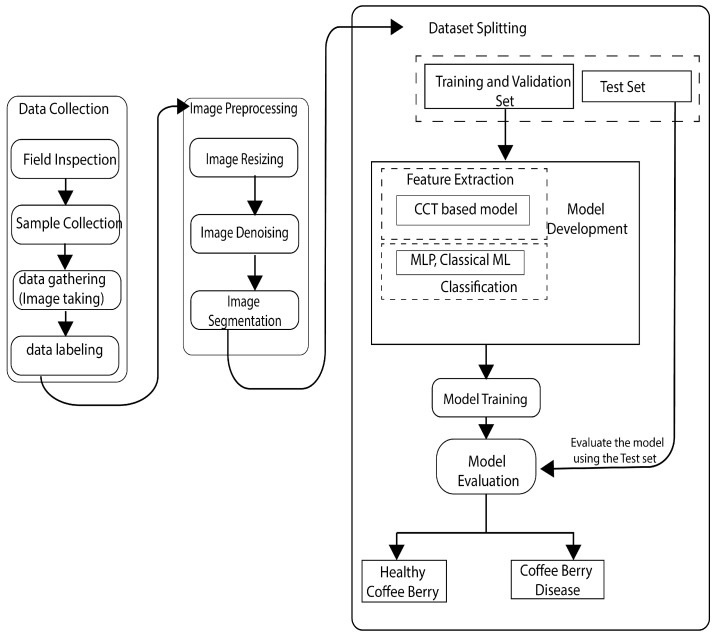
Proposed model architecture.

**Figure 3:**
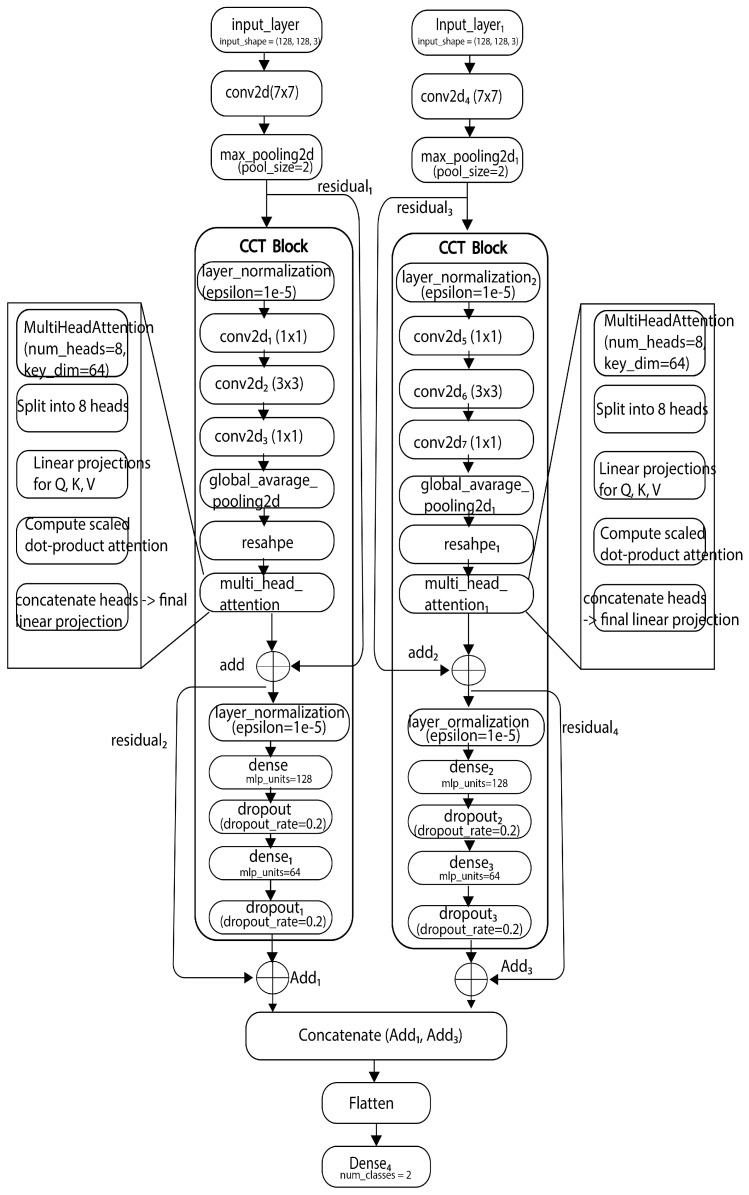
Proposed system model.

The proposed model architecture and model are illustrated in Figure 2 and Figure 3. Dataset splitting: The dataset was split into training, validation, and test sets using a stratified sampling strategy to ensure balanced class distributions across the sets. Training procedure: The model was trained using the training set with the Adam optimizer and binary crossentropy loss function. Hyperparameters such as learning rate, batch size, and number of epochs were fine-tuned through empirical experimentation to optimize model performance. Early stopping: To prevent overfitting, early stopping was employed based on the validation loss. Training was halted when the validation loss failed to decrease over a predefined number of epochs.

### 3.4. Deep Learning Architecture Used

Summary of the dual-input Convolutional Transformer model is shown in Table 1. The proposed model combines key elements of Convolutional Neural Networks (CNNs) and Compact Convolutional Transformers (CCTs). Specifically, we used convolutional layers for initial feature extraction and spatial encoding, followed by transformer-based attention mechanisms for capturing long-range dependencies. The convolutional tokenization method from CCT was employed instead of traditional patch embedding to preserve spatial locality. To enhance generalization and reduce overfitting, we integrated Sharpness-Aware Minimization (SAM) and applied layer normalization techniques within the transformer blocks. The final classification is performed using fully connected layers with softmax activation. This hybrid design leverages CNNs’ feature localization capability and transformers’ contextual understanding, making it suitable for agricultural disease classification tasks with limited training data.

### 3.5. The Architecture of Convolutional Network

Convolutional Neural Networks (CNNs) are deep learning architectures commonly used for image processing and computer vision tasks. Convolutional Neural Networks (CNNs) play a crucial role in applications like image classification, segmentation, and natural language processing. They consist of several layers, including the convolution layer, pooling layer, fully connected layer, and non-linear layer, each contributing to the network’s ability to learn and generalize [41]. Convolutional layers extract features by applying filters to detect patterns such as edges and textures [42]. Pooling layers reduce spatial dimensions, enhancing computational efficiency and controlling overfitting. These layers improve feature extraction robustness and facilitate faster training. Activation functions, such as ReLU, introduce non-linearity, addressing the vanishing gradient problem and enhancing learning efficiency. Fully connected layers integrate extracted features for final predictions, though they increase parameter count, requiring regularization techniques like dropout. Advances in CNNs, including ResNet and DenseNet, have improved accuracy and efficiency. Despite their success, CNNs face challenges in balancing accuracy and computational efficiency, driving ongoing research into advanced algorithms and novel design principles.

### 3.6. Model Concatenation

The proposed methodology utilizes a dual-input Compact Convolutional Transformer (CCT) model for coffee berry disease classification. Two parallel branches process input images through initial convolutional layers and a CCT block comprising convolutional layers, multi-head self-attention, layer normalization, and MLPs with residual connections. The output feature maps from these two branches are then concatenated to fuse the learned representations. This combined feature vector is subsequently processed by a global average pooling layer before being fed into a final dense output layer with a sigmoid activation for binary classification of healthy and CBD-affected coffee berries. This dual-pathway architecture, implemented using the TensorFlow Keras Functional API, aims to capture comprehensive features by processing the input through two parallel CCT streams before their integration for the final classification decision.

### 3.7. Evaluation Parameters

Evaluating deep learning models involves a comprehensive analysis of both functional and non-functional parameters to ensure their effectiveness and efficiency across various applications. The evaluation process typically includes assessing model performance through metrics like accuracy, sensitivity, specificity, and AUC, as well as considering computational efficiency and interpretability. These parameters are crucial for understanding a model’s generalization capabilities and suitability for specific tasks. The following sections detail the key evaluation parameters and techniques for deep learning models.

Accuracy:

Accuracy is the proportion of correct predictions made by the model out of all predictions made. It measures how well the model classifies the samples. The formula for accuracy is as follows:

$$Accuracy=\frac{TruePositives+TrueNegatives}{TotalSamples}$$

Recall (Sensitivity):

Recall is the fraction of positive instances that are correctly identified. It can be represented as follows:

$$Recall=\frac{True\;Positive}{True\;Positive+False\;Negative}$$

Precision:

Precision is the fraction of positive predictions that are actually correct. It can be represented as follows:

$$Precision=\frac{True\;Positive}{True\;Positive+False\;Positive}$$

where True Positives (TPs): are the number of instances that were correctly classified as positive by the model. True Negatives (TNs): The number of instances that were correctly classified as negative by the model. False Positives (FPs): The number of instances that were incorrectly classified as positive by the model. False Negatives (FNs): The number of instances that were incorrectly classified as negative by the model. Total Samples: The total number of instances in the dataset.

AUC:

Area Under the Curve (AUC) is a commonly used performance metric for machine learning classification problems, which evaluates the overall performance of a classifier. In binary classification, a classifier outputs a predicted probability for each sample to belong to one of two classes, positive or negative. The AUC is the area under the Receiver Operating Characteristic (ROC) curve, which plots the True Positive Rate (TPR) against the False Positive Rate (FPR) for different classification thresholds.

The equation for the ROC curve is as follows:

$$TPR=\frac{TP}{TP+FN}$$

$$FPR=\frac{FP}{FP+TN}$$

where TP, TN, FP, and FN are the number of True Positives, True Negatives, False Positives, and False Negatives, respectively.

The equation for the AUC can be written as follows:

$$AUC=\int_{-\infty }^{\infty }\left[\frac{TP}{TP+FN}-\frac{FP}{FP+TN}\right]ds$$

### 3.8. Hardware and Software Specifications

The experiments in this study were conducted using Google Colab’s free-tier environment (Google LLC, Mountain View, CA, USA), a cloud-based platform providing a Jupyter Notebook interface (version 6.5.7, Project Jupyter, global open-source community, sourced via Google Colab) with access to GPU acceleration. The hardware specifications include an Intel Xeon CPU (Intel Corporation, Santa Clara, CA, USA) at 2.20 GHz, an NVIDIA T4 Tensor Core GPU (NVIDIA Corporation, Santa Clara, California, USA, with HPE for integration in servers) (when enabled), 12 GB of RAM, and approximately 100 GB of temporary storage, subject to Colab’s limitations. The software environment is based on Ubuntu 20.04 (Canonical Ltd., London, UK), with Python 3.11.11 (Python Software Foundation, Wilmington, DE, USA) as the primary programming language. Machine learning frameworks utilized include TensorFlow 2.18.0 (Google Brain Team, Mountain View, CA, USA) and Keras 3.8.0 (Keras team, global open-source community). Additionally, Matplotlib (version 3.10.3, global open-source community, a NumFOCUS fiscally sponsored project) was used for model visualization, and Heapq (part of Python’s standard library, Python Software Foundation, Wilmington, DE, USA) was employed for heap queue operations. Google Colab’s dynamic cloud computing environment imposes session limitations, such as automatic resets and restricted runtimes, requiring periodic manual reconfiguration to maintain computational workflow continuity.

### 3.9. Classification

Following the feature extraction stage using the Compact Convolutional Transformer (CCT) model, the extracted feature vectors were fed into several machine learning classifiers for the task of classifying coffee berries into two categories: healthy and affected by coffee berry disease (CBD). The following classifiers were employed and their performance evaluated:

- **Multilayer Perceptron (MLP):** An MLP classifier with a Sigmoid activation function in the output layer was trained using the extracted CCT features. Early stopping was implemented based on the validation loss to prevent overfitting, with training halted if no improvement was observed for 12 consecutive epochs. Dropout (20%) was applied in the MLP layers for regularization, and layer normalization was used before each multi-head attention and MLP block within the CCT model to stabilize feature distributions.
- **Support Vector Machine (SVM):** An SVM classifier was utilized with hyperparameter tuning performed via Bayesian optimization. The search space for optimization included the regularization parameter C, kernel type (including polynomial), kernel coefficient, and polynomial degree. Five-fold cross-validation was employed during the optimization process over 50 iterations to identify the best hyperparameter configuration.
- **K-Nearest Neighbors (KNN):** The KNN classifier was optimized using Bayesian optimization to determine the optimal hyperparameters, including the distance metric (Manhattan), the number of nearest neighbors (k = 1), and the weighting scheme (distance-based). The optimization aimed to maximize the cross-validation score.
- **Decision Tree (DT):** Bayesian optimization was employed to find the best hyperparameters for the Decision Tree classifier. The optimized parameters included the splitting criterion (entropy), maximum depth, minimum samples per leaf, and minimum samples to split an internal node.
- **Random Forest (RF):** The Random Forest classifier was also optimized using Bayesian optimization. The hyperparameters tuned included the maximum depth of the trees, the maximum number of features to consider when looking for the best split, the minimum number of samples required to be at a leaf node, the minimum number of samples required to split an internal node, and the number of trees (estimators) in the forest.

For each classifier, the performance was evaluated on a held-out test set using metrics such as accuracy and, where applicable, the Area Under the Receiver Operating Characteristic curve (AUC). Confusion matrices were also analyzed to gain insights into the model’s ability to correctly classify each category and identify any patterns of misclassification. The hyperparameters identified through Bayesian optimization for each classifier were used for the final evaluation on the test set.

This multi-classifier approach aimed to provide a comprehensive evaluation of the features extracted by the proposed CCT model and to identify the most effective classification strategy for the automated detection of coffee berry disease.

## 4. Results

The Results section provides an in-depth analysis of our proposed approach’s performance. We evaluate key performance metrics, including training and validation accuracy as well as loss. The confusion matrix is utilized to examine the behavior of distinct classes, offering insights into classification performance. Additionally, ROC curve plots are generated to visualize the model’s performance at various threshold levels. To contextualize our findings, we compare our results with other state-of-the-art approaches, highlighting the strengths and advantages of our proposed method.

### 4.1. Results After Segmentation and Noise Removal

To assess the impact of bilateral filtering and color segmentation on classification performance, we evaluated multiple machine learning models before and after applying these preprocessing techniques. The results demonstrate a consistent improvement across all classifiers, highlighting the effectiveness of these techniques in enhancing feature quality and overall model performance.

We selected bilateral filtering as the primary denoising technique, particularly for the Compact Convolutional Transformer (CCT) model, due to its capability to reduce noise while preserving important edge details in RGB images. Unlike other conventional denoising methods such as Gaussian or median filtering, which often blur both noise and important image structures, bilateral filtering considers both the spatial proximity and intensity differences between pixels. This characteristic is particularly beneficial in our case because the initial convolutional layers of the CCT architecture are designed to extract local spatial features such as edges and textures, key elements for distinguishing between healthy and diseased coffee berries. Preserving these details ensures that subsequent transformer layers receive more structurally meaningful features, which can enhance the model’s ability to capture long-range dependencies. While methods like non-local means filtering are also effective for denoising, they tend to be computationally expensive and may introduce artifacts or over smoothing in cases where subtle texture differences are crucial for classification. In contrast, bilateral filtering offers a well balanced approach, achieving good noise suppression with edge preservation at a reasonable computational cost.

To identify the optimal parameters for bilateral filtering, specifically the spatial diameter and the σ values for both the spatial and color (intensity) domains, we employed Bayesian optimization instead of relying on empirical experimentation. This method allowed for an efficient exploration of the parameter space by treating the classification accuracy of the MLP model on the validation set as the objective function. Through iterative updates guided by a surrogate model, Bayesian optimization was able to converge on the optimal parameter values: a spatial diameter (*d*) of 9, $\sigma_{\mathrm{color}}=75$, and $\sigma_{\mathrm{space}}=75$. This automated search strategy proved to be more effective and computationally efficient compared to manual tuning or exhaustive grid search. The selected parameters improved both the visual quality of the filtered images and the performance of the classifiers, ensuring that important structural features were retained without introducing excessive smoothing artifacts.

Before applying bilateral filtering and color segmentation, the Multilayer Perceptron (MLP) achieved an accuracy of 86.61%, which increased to 91.28% after preprocessing. Similarly, the Support Vector Machine (SVM) showed an improvement from 75.61% to 79.7%, while the K-Nearest Neighbors (KNN) classifier improved from 85.61% to 88.13%. The Decision Tree (DT) classifier experienced an accuracy boost from 87.61% to 90.72%, and the Random Forest (RF) classifier improved from 88.61% to 91.14%.

**Figure 4:**
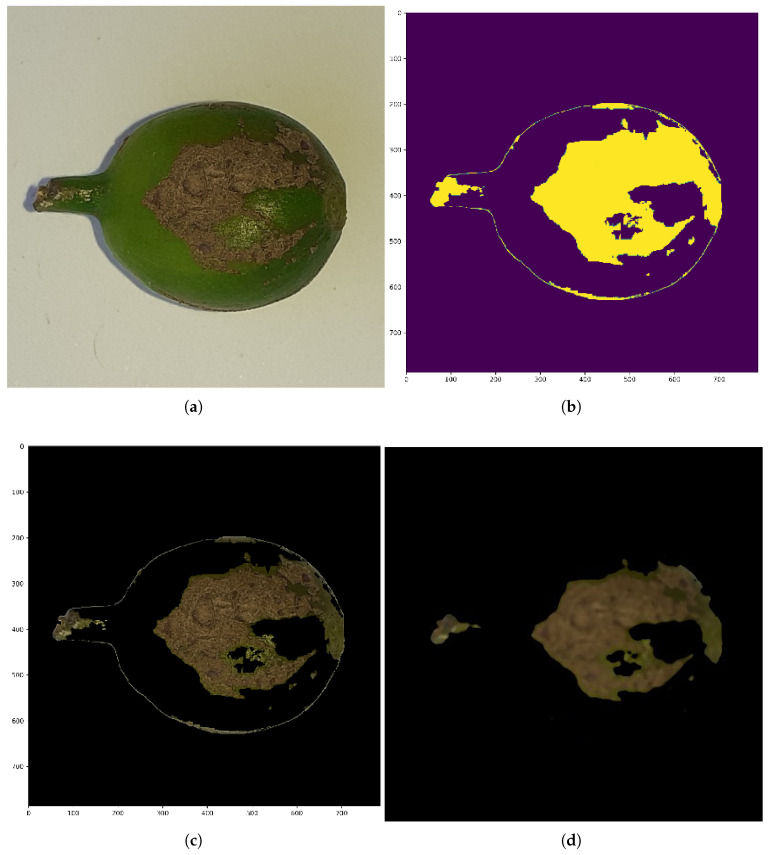
Illustration of the segmentation process applied to a sample image. (**a**) Original coffee berry image. (**b**) Extracted mask highlighting the region of interest. (**c**) Initial segmented image showing the isolated berry area. (**d**) Final segmented image after applying post-processing steps such as noise removal, erosion, and dilation to enhance accuracy and clarity.

These results, shown in Table 2, indicate that bilateral filtering effectively smooths noise while preserving edge details, leading to enhanced feature extraction. Meanwhile, color segmentation refines object boundaries, ensuring that classifiers receive more discriminative input features (see Figure 4). The overall improvement in accuracy across all models confirms that these preprocessing techniques contribute to better representation learning, ultimately leading to enhanced classification performance.

### 4.2. Results After Identifying Optimal Hyper Parameters

This section details the performance of each machine learning classifier after undergoing hyperparameter optimization using Bayesian optimization. The specific hyperparameters that yielded the best performance for each model are presented in Table 3, followed by a detailed report of the results obtained with these optimized settings.

#### 4.2.1. MLP

The Compact Convolutional Transformer (CCT) with Sigmoid activation was trained using early stopping to optimize performance while preventing overfitting. We used an early stopping mechanism with a patience value of five epochs, meaning training stops if validation loss does not improve for five consecutive epochs. This value was chosen empirically based on multiple trials to prevent overfitting while allowing sufficient time for convergence. After 103 epochs, the model achieved a training accuracy of 98.96% and a validation accuracy of 97.70%, with a validation loss of 0.0526, indicating strong generalization. The model incorporates multiple regularization techniques to enhance robustness, including dropout (20%) in the MLP layers, which prevents overfitting by randomly deactivating neurons during training. Additionally, layer normalization is applied before each multi-head attention and MLP block, ensuring stable feature distributions and improved convergence. Early stopping also ensures training efficiency by restoring the best-performing model weights and avoiding unnecessary computations.

**Figure 5:**
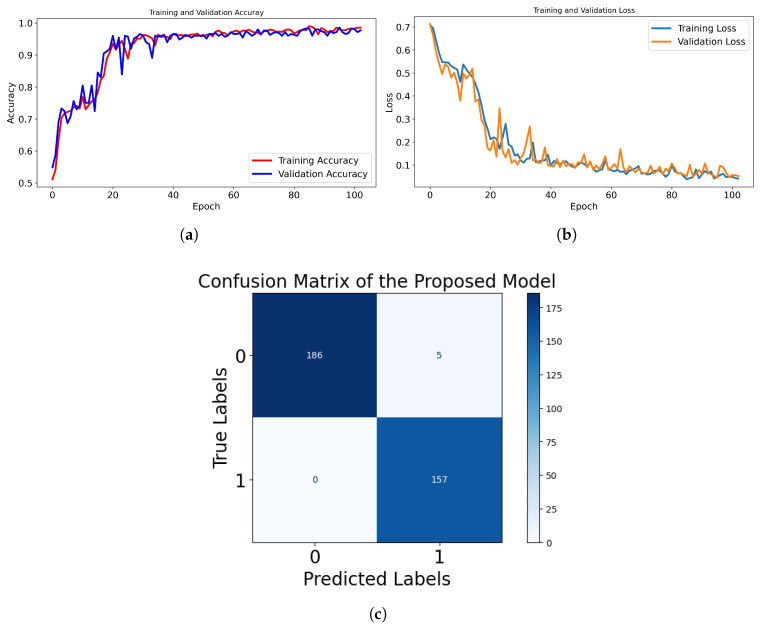
Performance evaluation of the proposed model using the MLP classifier. (**a**) Training and validation accuracy curve showing the model’s learning progression over epochs. (**b**) Training and validation loss curve indicating convergence behavior. (**c**) Confusion matrix illustrating classification results, where label 0 represents healthy coffee berries, and label 1 denotes diseased coffee berries.

As shown in Figure 5c, the confusion matrix analysis further highlights the model’s strong performance. It correctly classified 186 healthy samples and 157 CBD samples, with only 5 healthy samples misclassified as CBD and 0 CBD samples misclassified as healthy. The 100% sensitivity for CBD detection ensures that no CBD cases go undetected.

#### 4.2.2. SVM

**Figure 6:**
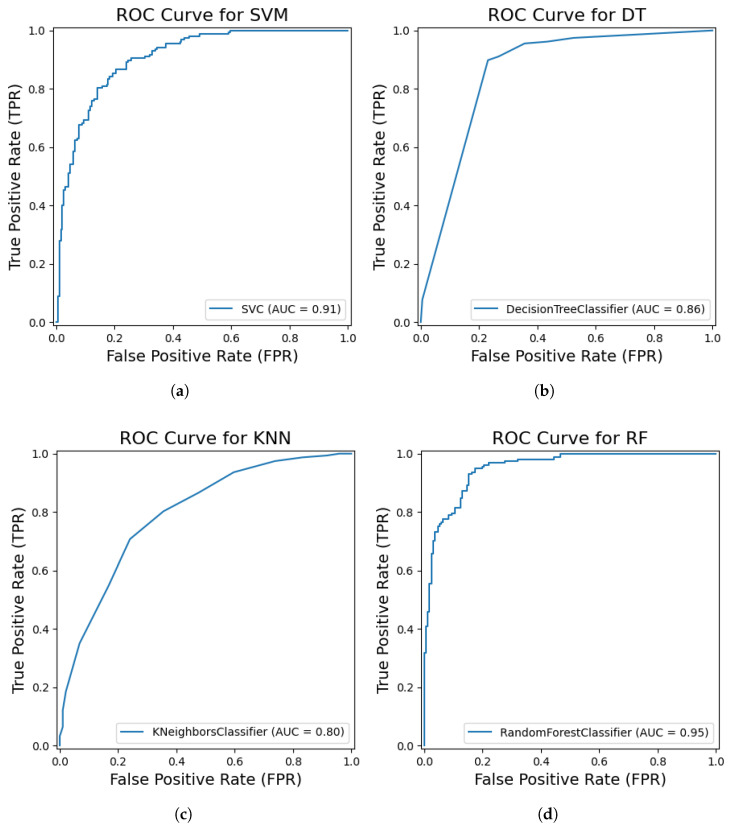
Receiver Operating Characteristic (ROC) curves and Area Under the Curve (AUC) scores for various classical machine learning classifiers applied to coffee berry classification. (**a**) ROC curve and AUC for Support Vector Machine (SVM). (**b**) ROC curve and AUC for Decision Tree (DT). (**c**) ROC curve and AUC for K-Nearest Neighbors (KNN). (**d**) ROC curve and AUC for Random Forest (RF). These plots illustrate the discriminative ability of each model across different classification thresholds.

To evaluate the effectiveness of the Compact Convolutional Transformer (CCT) as a feature extractor, a Support Vector Machine (SVM) classifier was employed and optimized using Bayesian optimization. The best-performing SVM model, identified after 50 iterations of Bayesian optimization with five-fold cross-validation, utilized the following hyperparameters: C=3.88, polynomial kernel with γ=1.0, and degree=5. This optimized SVM classifier achieved a test set accuracy of 82.47%, with a precision of 0.83, recall of 0.82, F1-Score of 0.83, and an AUC score of 0.91 (Figure 6a). The confusion matrix revealed 156 correctly classified healthy samples and 131 correctly classified CBD samples, alongside 35 healthy samples misclassified as CBD and 26 CBD samples misclassified as healthy. The performance metrics indicate a robust classification capability achieved through the synergy of CCT-extracted features and an optimized SVM.

#### 4.2.3. KNN

The K-Nearest Neighbors (KNN) classifier was optimized using Bayesian optimization, which identified the best hyperparameters as the Manhattan distance metric, one nearest neighbor, and distance-based weighting. When evaluated on the test set, the KNN model achieved an accuracy of 73.6%, with a precision of 0.74, a recall of 0.74, an F1-Score of 0.74, and an AUC score of 0.80 (see Figure 6c). The confusion matrix analysis showed that 145 healthy samples were correctly classified, while 46 healthy samples were misclassified as CBD. Similarly, 46 CBD samples were correctly classified, whereas 111 CBD samples were misclassified as healthy. The optimized KNN model demonstrates a moderate classification performance.

#### 4.2.4. DT

The Decision Tree classifier was optimized using Bayesian optimization, which identified the best hyperparameters as entropy as the splitting criterion, a maximum depth of 30, a minimum of one sample per leaf, and a minimum split of two samples. The model achieved a test accuracy of 82.76%, along with a precision of 0.84, a recall of 0.83, and an F1-Score of 0.83. As shown in Figure 6b, the model achieved an AUC score of 0.86, indicating a good ability to distinguish between the classes. The confusion matrix analysis showed that 147 healthy samples were correctly classified, while 44 healthy samples were misclassified as CBD. Similarly, 16 CBD samples were correctly classified, whereas 141 CBD samples were misclassified as healthy. The optimized Decision Tree classifier demonstrates a competitive performance.

#### 4.2.5. RF

**Figure 7:**
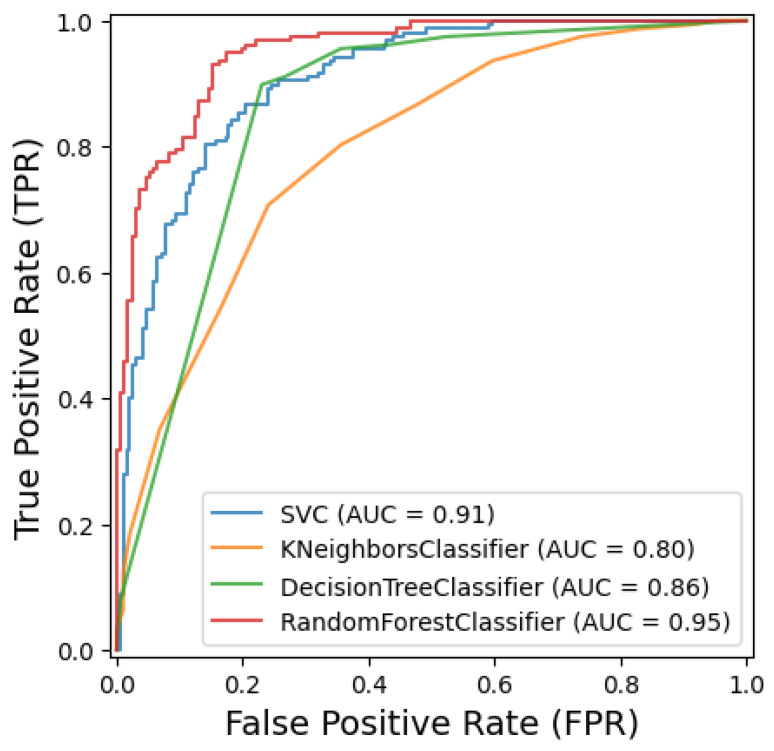
Comparative results of machine learning algorithms.

The Random Forest classifier was optimized using Bayesian optimization, which identified the best hyperparameters as a maximum depth of 43, ‘log2’ as the maximum number of features considered per split, a minimum of one sample per leaf, a minimum split of three samples, and 222 estimators (trees) in the ensemble. When tested on the data, the model achieved a test accuracy of 73.56%, along with a precision of 0.76, a recall of 0.74, and an F1-Score of 0.72. As shown in Figure 6d and Figure 7, the AUC score of 0.95 indicates a strong ability to distinguish between classes. A breakdown of the confusion matrix reveals that 175 healthy samples were correctly classified, while 16 healthy samples were misclassified as CBD. On the other hand, the model correctly classified 81 CBD samples, with 76 misclassified as healthy. The Random Forest model demonstrates moderate accuracy but a strong ability to distinguish between the two classes based on the AUC score.

### 4.3. Results of Machine Learning Classifiers

### 4.4. Robustness Analysis Under Low-Resolution and Occlusion Conditions

To assess the robustness of our proposed model under adverse conditions, we conducted experiments involving two types of image degradations: low-resolution input and occlusion-based noise. These scenarios simulate real world challenges such as poor imaging conditions, compression artifacts, or damaged samples.

**Low-Resolution Evaluation:** To evaluate the effect of reduced input resolution, the original 128 × 128 images were downsampled to 64 × 64 and 32 × 32 pixels using bicubic interpolation. This was achieved by applying a scale factor of 0.5 for 64 × 64 and 0.25 for 32 × 32. After downsampling, the images were upsampled back to 128 × 128 using the same bicubic interpolation method before being fed into the model. This simulates real-world situations where low-quality or bandwidth-constrained images are received and resized for standard model input dimensions. At 64 × 64 (scale factor = 0.5), the model retained much of its performance, showing minimal drop in accuracy. At 32 × 32 (scale factor = 0.25), a more noticeable degradation occurred, but the model still performed reasonably, indicating good generalization from global structures.

**Occlusion Noise Evaluation:** To simulate occlusion scenarios, we applied two types of noise: *Gaussian noise* (μ=0, σ=0.05)—introduced uniformly across all pixels. *Random block occlusion:* black rectangular regions of size 25 × 25 pixels were randomly placed over the berry area to simulate leaf shadow, fruit overlap, or annotation errors. These techniques aimed to test the model’s ability to make correct predictions even when part of the informative region is distorted or missing.

The experimental results demonstrate that while performance degrades slightly in both conditions, the model maintains a high classification accuracy—particularly under mild resolution loss or light occlusion. This robustness can be attributed to the transformer’s ability to capture global context and the effectiveness of our preprocessing pipeline (bilateral filtering + segmentation) in highlighting salient features, as summarized in Table 4.

### 4.5. Comparative Results of CCT with Known Pretrained Models

To evaluate the performance of our proposed model, we conducted a comparative analysis against several well-established pretrained architectures, including AlexNet, VGG, ResNet, EfficientNet, and MobileViT. The comparison was based on three key performance metrics: classification accuracy, the number of trainable parameters, and average training time per epoch. Table 5 presents a summary of the results.

The results clearly show that the proposed model achieves the highest classification accuracy (97.5%), outperforming all the compared architectures, including MobileViT (93.28%) and ResNet (91.41%). This underscores the model’s effectiveness in capturing and learning relevant features, enabling precise identification of coffee berry diseases.

Moreover, the proposed model demonstrates exceptional computational efficiency. It has the fewest trainable parameters (0.408 million), significantly lower than AlexNet (60 million), VGG (138 million), and even the lightweight MobileViT (6.4 million). This compact architecture translates to reduced memory requirements and faster computations.

In terms of training time, the proposed model again proves advantageous, requiring only 2.3 s per epoch. In contrast, MobileViT requires 33.7 s, and VGG requires as much as 103 s per epoch. The rapid training speed makes the proposed model especially suitable for deployment in real-time and resource-constrained agricultural settings.

This comparative analysis highlights that the proposed model not only delivers superior classification performance but also achieves an optimal balance between accuracy, parameter efficiency, and training speed. These qualities make it a compelling solution for practical, real-world applications in smart agriculture and precision farming.

## 5. Discussion

The experimental findings underscore the effectiveness of our proposed approach, particularly the integration of preprocessing techniques, Compact Convolutional Transformer (CCT) for feature extraction, and various optimized classifiers. The use of bilateral filtering and color segmentation as a preprocessing step resulted in significant performance gains across all classifiers, affirming their ability to enhance the discriminative power of image features. Notably, improvements were most pronounced in models like MLP.

The CCT model, when paired with a Multilayer Perceptron (MLP) classifier, demonstrated the highest classification performance with a validation accuracy of 97.70% and a validation loss of only 0.0526. The confusion matrix revealed excellent sensitivity for CBD detection (100%), making it particularly valuable in applications where false negatives carry high risks, such as agricultural disease diagnosis or medical imaging. The integration of dropout, layer normalization, and early stopping not only improved generalization but also prevented overfitting, which is critical for real-world deployment.

This result is consistent with the findings of [24], where CoffeeNet achieved 98.54% accuracy using a ResNet-50-based attention mechanism, and [8], which reported 96% accuracy with EfficientNetB0. Moreover, the 100% sensitivity for CBD detection in our CCT-MLP model outperforms the Deep-Siamese CNN approach reported in [22], which achieved 96.89% accuracy. These comparisons highlight the superior ability of the CCT architecture to capture relevant spatial and contextual information, especially when integrated with MLP. Among traditional classifiers, the optimized DT model delivers a competitive result with a test accuracy of 82.76% and an AUC of 0.86. The SVM also shows a strong performance with an accuracy of 82.47% and a high AUC of 0.91. The KNN model achieved an accuracy of 73.6% and an AUC of 0.80, while the RF model achieved an accuracy of 73.56% with an AUC of 0.95. The confusion matrices for these models reveal varying patterns of misclassification. SVM and DT exhibit a relatively balanced performance, while KNN shows a higher number of misclassifications for CBD as healthy. The RF model, despite a high AUC, shows a notable number of false negatives for CBD.

The results indicate that the CCT-extracted features provide a robust basis for classification across various machine learning models. The CCT-MLP combination demonstrates the strongest performance in terms of accuracy and sensitivity. Traditional classifiers like SVM and DT also achieve competitive results. The performance of KNN and RF suggests that while the features are informative, these models might be more sensitive to the specific characteristics of the test data.

The synergy between advanced preprocessing, transformer-based feature extraction, and model-specific hyperparameter tuning leads to substantial gains in classification accuracy and reliability. The pipeline’s modular design also facilitates adaptability to other datasets and classification problems beyond coffee berry disease.

## 6. Conclusions

This study introduced a robust image classification pipeline leveraging advanced preprocessing and Compact Convolutional Transformer (CCT) for feature extraction, coupled with optimized machine learning classifiers. The key finding is that bilateral filtering and color segmentation significantly improved feature quality, leading to enhanced classification accuracy across all models. Notably, the CCT-MLP architecture achieved the highest validation accuracy of 97.70% and exceptional sensitivity for CBD detection, demonstrating the pipeline’s potential for accurate classification. The effectiveness of CCT-extracted features was further validated by the strong performance of optimized SVM and DT classifiers. These findings highlight the pipeline’s promise for applications in agriculture and medical diagnostics. Future research will focus on real-time implementation, multi-class classification, IoT integration, dataset expansion, transfer learning, and in-depth analysis of classifier performance with CCT features for application-specific optimization.

## Figures and Tables

**Table 1:** Summary of the dual-input Convolutional Transformer model. Overview of the model’s architecture, including layer details, output shapes, and parameter counts. It processes two input images simultaneously, applying convolutional layers followed by a custom CCT block to capture spatial and global dependencies. The outputs are then merged, flattened, and passed through a final dense layer for classification.

Layer (Type)	Output Shape	Param #	Connected to
input_layer	(None, 128, 128, 3)	0	–
input_layer_1_	(None, 128, 128, 3)	0	–
conv2d	(None, 128, 128, 64)	9472	input_layer
conv2d_4_	(None, 128, 128, 64)	9472	input_layer_1_
max_pooling2d	(None, 64, 64, 64)	0	conv2d
max_pooling2d_1_	(None, 64, 64, 64)	0	conv2d_4_
layer_normalization	(None, 64, 64, 64)	128	max_pooling2d
layer_normalization_2_	(None, 64, 64, 64)	128	max_pooling2d_1_
conv2d_1_	(None, 64, 64, 64)	4160	layer_normalization
conv2d_5_	(None, 64, 64, 64)	4160	layer_normalization_2_
conv2d_2_	(None, 64, 64, 64)	36,928	conv2d_1_
conv2d_6_	(None, 64, 64, 64)	36,928	conv2d_5_
conv2d_3_	(None, 64, 64, 64)	4160	conv2d_2_
conv2d_7_	(None, 64, 64, 64)	4160	conv2d_6_
global_average_pooling2d	(None, 64)	0	conv2d_3_
global_average_pooling2d_1_	(None, 64)	0	conv2d_7_
reshape	(None, 1, 64)	0	global_average_pooling2d
reshape_1_	(None, 1, 64)	0	global_average_pooling2d_1_
multi_head_attention	(None, 1, 64)	132,672	reshape, reshape
multi_head_attention_1_	(None, 1, 64)	132,672	reshape_1_, reshape_1_
add	(None, 64, 64, 64)	0	max_pooling2d, multi_head_attention
add_2_	(None, 64, 64, 64)	0	max_pooling2d_1, multi_head_attention_1_
layer_normalization_1_	(None, 64, 64, 64)	128	add
layer_normalization_3_	(None, 64, 64, 64)	128	add_2_
dense	(None, 64, 64, 128)	8320	layer_normalization
dense_2_	(None, 64, 64, 128)	8320	layer_normalization
dropout_1_	(None, 64, 64, 128)	0	dense
dropout_4_	(None, 64, 64, 128)	0	dense_2_
dense_1_	(None, 64, 64, 64)	8256	dropout_1_
dense_3_	(None, 64, 64, 64)	8256	dropout_4_
dropout_2_	(None, 64, 64, 64)	0	dense_1_
dropout_5_	(None, 64, 64, 64)	0	dense_3_
add_1_	(None, 64, 64, 64)	0	add, dropout_2_
add_3_	(None, 64, 64, 64)	0	add_2_, dropout_5_
concatenate	(None, 64, 64, 128)	0	add_1_, add_3_
flatten	(None, 524,288)	0	concatenate
dense_4_	(None, 2)	1,048,578	flatten

**Table 2:** Classification accuracy before and after applying bilateral filtering and color segmentation. The results demonstrate the impact of these preprocessing steps on enhancing the model’s ability to accurately distinguish between healthy and diseased coffee berries.

Classifier	Accuracy Before (%)	Accuracy After (%)
Multilayer Perceptron (MLP)	86.61	91.28
Support Vector Machine (SVM)	75.61	79.70
K-Nearest Neighbors (KNN)	85.61	88.13
Decision Tree (DT)	87.61	90.72
Random Forest (RF)	88.61	91.14

**Table 3:** Optimal hyperparameters for machine learning classifiers.

Classifier	Hyperparameter	Optimal Value
SVM	C (Regularization)	3.88
	Kernel Type	Polynomial
	γ (Kernel Coefficient)	1.0
	Degree (Polynomial Kernel)	5
KNN	Distance Metric	Manhattan
	Number of Neighbors (K)	1
	Weighting	Distance
DT	Splitting Criterion	Entropy
	Maximum Depth	30
	Minimum Samples per Leaf	1
	Minimum Samples to Split	2
RF	Maximum Depth	43
	Maximum Features	log2
	Minimum Samples per Leaf	1
	Minimum Samples to Split	3
	Number of Estimators	222

**Table 4:** Model robustness under low-resolution and occlusion settings. Accuracy drops are shown relative to the original high-resolution clean image performance.

Condition	Test Accuracy (%)	Drop from Original (%)
Original (128 × 128, no noise)	97.70	0.00
Low Resolution (64 × 64, scale = 0.5)	95.42	−2.28
Low Resolution (32 × 32, scale = 0.25)	91.35	−6.35
Gaussian Noise (σ=0.05)	92.80	−4.90
Random Block Occlusion (25 × 25 px)	89.62	−8.08

**Table 5:** Comparison between the proposed model and various pretrained deep learning models based on classification accuracy, number of trainable parameters, and training time per epoch. This evaluation demonstrates the effectiveness and computational efficiency of the proposed model in classifying coffee berry disease.

Model	Accuracy (%)	Parameters (Millions)	Time (s/Epoch)
AlexNet	83.70	60.0	42.0
VGG	87.05	138.0	103.0
ResNet	91.41	25.6	58.0
EfficientNet	90.46	30.6	61.0
MobileViT	93.28	6.4	33.7
**Proposed Model**	**97.50**	**0.408**	**2.3**

## Data Availability

Data will be available upon reasonable request.

## Abbreviations

The following abbreviations are used in this manuscript:

MLP	Multilayer Perceptron
KNN	K-Nearest Neighbors
SVM	Support Vector Machine
RF	Random Forest
DT	Decision Tree
CBD	Coffee Berry Disease
CCT	Compact Convolution Transformer
